# Pasakbumin A controls the growth of *Mycobacterium tuberculosis* by enhancing the autophagy and production of antibacterial mediators in mouse macrophages

**DOI:** 10.1371/journal.pone.0199799

**Published:** 2019-03-13

**Authors:** Hyo-Ji Lee, Hyun-Jeong Ko, Seung Hyun Kim, Yu-Jin Jung

**Affiliations:** 1 Department of Biological Sciences and Institute of Life Sciences, Kangwon National University, Chuncheon, Republic of Korea; 2 College of Pharmacy, Kangwon National University, Chuncheon, Republic of Korea; 3 College of Pharmacy, Yonsei University, Incheon, Republic of Korea; Universita Cattolica del Sacro Cuore, ITALY

## Abstract

Tuberculosis (TB) is a chronic infectious disease caused by *Mycobacterium tuberculosis* (Mtb) and remains a major health problem worldwide. Thus, identification of new and more effective drugs to treat emerging multidrug-resistant TB (MDR-TB) and to reduce the side effects of anti-TB drugs, such as liver toxicity and other detrimental changes, is urgently needed. In this study, to develop a novel candidate drug for effective TB treatment with few side effects in the host, we selected pasakbumin A isolated from *Eurycoma longifolia* (*E*. *longifolia*) Jack, which protected host cells against Mtb infection-induced death. Pasakbumin A significantly inhibited intracellular Mtb growth by inducing the autophagy via the ERK1/2-mediated signaling pathway in Mtb-infected macrophages. We further investigated whether pasakbumin A could be used as a potential adjuvant for TB treatment. Treatment with pasakbumin A and anti-TB drug rifampicin (RMP) potently suppressed intracellular Mtb killing by promoting autophagy as well as TNF-α production via the ERK1/2- and NF-κB-mediated signaling pathways in Mtb-infected cells. Our results suggest that pasakbumin A could be developed as a novel anti-TB drug or host-directed therapeutic (HDT) strategy to protect against host cell death and improve host defense mechanisms against Mtb infection in macrophages.

## Introduction

Tuberculosis (TB) is still one of the oldest known human diseases and a major cause of mortality among the infectious diseases[1]. *Mycobacterium tuberculosis* (Mtb), the causative agent of TB, is a highly successful facultative intracellular pathogen that can persist within host phagocytes[2]. Mtb infection usually begins after inhalation of aerosol droplets that contain bacteria into the pulmonary alveoli. After inhalation, Mtb is recognized by resident alveolar macrophages, dendritic cells and recruited monocytes through various pattern recognition receptors (PRRs)[3]. These receptors initiate diverse signal transduction pathways, including the nuclear factor-kappa B (NF-κB) and mitogen-activated protein kinase (MAPK) signaling pathways, which induce the production of cytokines and chemokines in host cells[4]. Induction of these effector molecules regulates bacterial growth and promotes the adaptive immune response. Mtb is also ingested by phagocytosis to form phagosome containing Mtb-antigen (Mtb-Ag). After phagocytosis, mycobacterial antigens are processed and presented to Mtb-specific CD4^+^ T cells and CD8^+^ T cells, which produce several cytokines to activate macrophages and lymphocytes[5]. However, Mtb can survive and persist inside macrophages in the dormant stage for a long period by interfering with the host immune system to avoid elimination by the effector immune cells[6, 7].

Autophagy is a conserved lysosomal self-digestion process that involves turnover of cellular constituents to maintain cellular homeostasis[8]. This process also functions as an innate immune defense mechanism against infectious pathogens through the fusion of the lysosome with a double-membrane-bound autophagosome, which can sequester cytoplasmic materials and pathogens[9, 10]. The autophagic process is tightly regulated by the action of autophagy-related (Atg) proteins, such as beclin-1 and microtubule-associated protein 1A/1B-light chain 3 (LC3)[11, 12]. Because a cytosolic LC3 (LC3-I) is conjugated with phosphatidylethanolamine (PE) to form membrane-bound lapidated LC3 (LC3-II) during autophagy[13], the conversion of LC3-I to LC3-II is commonly used to measure and monitor autophagy. However, Mtb has various mechanisms for evasion of innate immune system. Mtb secretes an enhanced intracellular survival (Eis) protein which inhibits autophagy by increasing IL-10 expression[14]. This mechanism plays a role as innate immune response evasion mechanism. Although many studies have shown that the activation of autophagy not only enhances phagosome-lysosome fusion but also regulates Mtb growth in host cells[15], Mtb has evolved several mechanisms to modulate or exploit the autophagic process[16–18].

Current TB treatment is based on multidrug chemotherapy. According to the WHO guide lines, a multidrug regimen for TB includes administration of first-line drugs consisting of rifampicin (RMP), isoniazid (INH), pyrazinamide (PZA), and ethambutol (EMB) for 2 months followed by INH and RMP for 4 months[19]. However, prolonged regimens using the same few drugs have resulted in poor patient compliance which leads to the emergence of strains with resistant to the available anti-TB drugs, including multidrug (MDR) and extensively drug resistant (XDR) Mtb[20–22]. Due to the increased emergence of drug-resistant Mtb strains, there in an urgent need for the development of new anti-TB drugs. Recently, attention has focused on a new and emerging concept in the treatment of TB known as host-directed therapy (HDT), which focuses on key components of host anti-mycobacterial effector mechanisms and limiting inflammation and tissue damage[23–25]. Therefore, in this study, we identified a novel anti-TB drug from natural compounds that exhibited antibacterial activity by enhancing host anti-TB effector mechanisms in mouse macrophages. To screen the anti-Mtb activities of selected natural compounds, we measured the bacterial growth in Mtb-infected macrophages after treatment with each compound. The best candidates among the selected natural compounds was pasakbumin A isolated from *Eurycoma longifolia* (*E*. *longifolia*) Jack, which is commonly used as a traditional herbal medicine to treat fever, malaria, ulcers and TB[26]. We observed significant inhibition of intracellular Mtb growth via induction of autophagy and increased production of NO and TNF-α in pasakbumin A-treated cells during Mtb infection. In addition, the combined treatment of the anti-TB drug RMP and pasakbumin A strongly reduced intracellular Mtb growth by promoting autophagy and inflammatory cytokine production via the ERK1/2- and NF-κB-mediated pathways in Mtb-infected cells. Together, our results suggest that pasakbumin A can enhance the Mtb-killing activity of macrophages by inducing autophagy. Pasakbumin A could be developed as a HDT drug and/or therapeutic strategy that enhances innate immune functions and modulates inflammatory responses for TB.

## Results

### Pasakbimin A controls intracellular Mtb growth by increasing the production of TNF-α and NO in Mtb-infected macrophages

**Fig 1:**
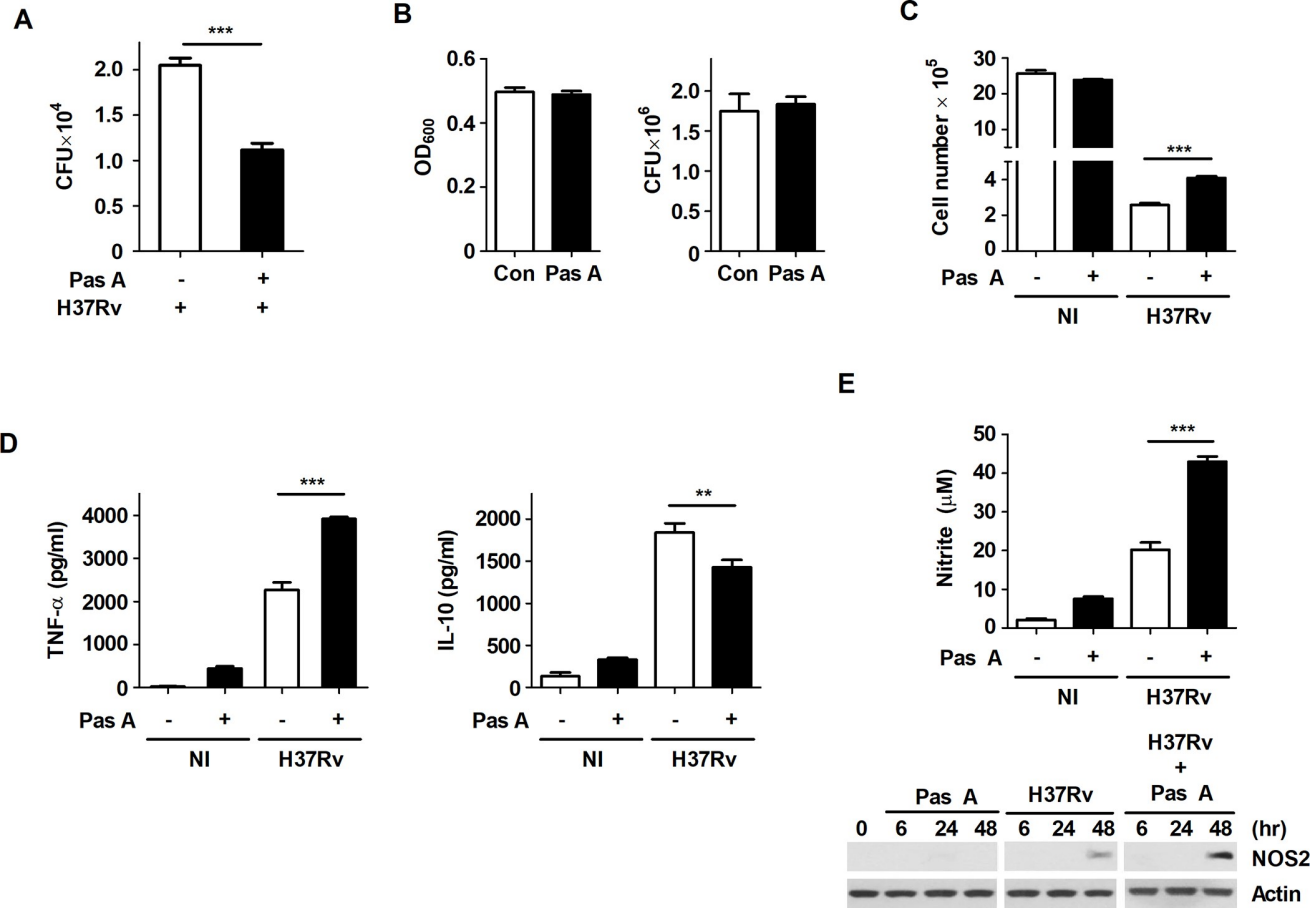
Pasakbumin A controls intracellular Mtb growth by increasing the production of NO and pro-inflammatory cytokine in H37Rv-infected macrophages. Raw264.7 macrophages were stimulated with pasakbumin A (Pas A, 10 μM) for 48 h after infection with H37Rv (at MOIs of 1 or 5). (A) Intracellular bacterial survival was determined by counting the number of CFUs at 3-weeks after inoculation. (B) The cultures were grown in 7H9 medium supplemented with 10% ADC containing 0.2% glycerol at 37°C for 72 h with or without pasakbumin A (Pas A, 10 μM). Left, bacterial growth was measured as OD_600_ at 72 h after Mtb inoculation. Right, colony-forming units (CFUs) were measured by plating bacterial dilutions onto 7H10 agar supplemented with 10% OADC containing 0.5% glycerol at 72 h. Error bars represents the standard deviation of the mean. (C) Cell viability was assessed using trypan-blue exclusion assay. (D) Culture supernatants were measured for the production of TNF-α and IL-10 by ELISA in non-infected (NI) or H37Rv-infected cells. (E) Bar graph in the left panel represents NO production (as indicated by the nitrite level) by diazotization (Griess method) assay. Right panel represents NOS2 protein by western blot analysis. Actin served as a loading control. The full length of all western blots has been provided in the **S1 Fig**. Statistical significance is indicated as **, *p*<0.01 and ***, *p*<0.001.

Recent studies have shown that various compounds extracted from leaves, stem and root of *E*. *longifolia* exhibit potent antibacterial activity against pathogenic Gram-positive and Gram-negative bacteria[27]. However, the specific compounds extracted from *E*. *longifolia* that control intracellular Mtb growth remain poorly understood, although *E*. *longifolia* is also used as a traditional medicine for patients with TB in Malaysia. To identify the mycobactericidal activity of the compounds extracted from *E*. *longifolia* in macrophages, we assessed intracellular bacterial survival in H37Rv-infected cells treated with each compound. We obtained a specific compound, pasakbumin A, with potential mycobactericidal activities against Mtb infection. Pasakbumin A significantly suppressed intracellular Mtb growth in macrophages (**S3C Fig and Fig 1A**), although it was not directly toxic to Mtb (**Fig 1B**). Recently, Tousif *et al*. showed that the anti-TB drug INH strongly induces apoptosis of activated CD4^+^ T cells and reduces Mtb antigen-specific immune responses[28], suggesting that anti-TB drugs induce host cell damage as well as mycobacterial killing. In contrast to most commonly used anti-TB drugs, pasakbumin A was sufficient to protect Mtb-infected Raw264.7 cells compared to cells infected with Mtb alone, and it did not exhibit any cytotoxic effect in macrophages (**Fig 1C**). To further clarify whether pasakbumin A prevents host cell death during Mtb infection, cell death was measured by Annexin-V/PI staining or LDH assay. We investigated that pasakbumin A decreased necrotic and apoptotic cell population (**S3A Fig**) as well as LDH release (**S3B Fig**) during Mtb infection. In addition, TNF-α production substantially increased in pasakbumin A-treated Raw264.7 macrophages during Mtb infection, but IL-10 production significantly decreased (**Fig 1D**). Notably, we found that pasakbumin A strongly enhanced NO production and NOS2 expression in Raw264.7 macrophages during Mtb infection compared with those in Mtb-infected cells (**Fig 1E**).

These results suggest that pasakbumin A controls intracellular Mtb growth by enhancing the production of NO and TNF-α in macrophages and protects against host cell death during Mtb infection.

### Pasakbumin A activates the ERK1/2-mediated signaling pathway and induces autophagy in Mtb-infected macrophages

**Fig 2:**
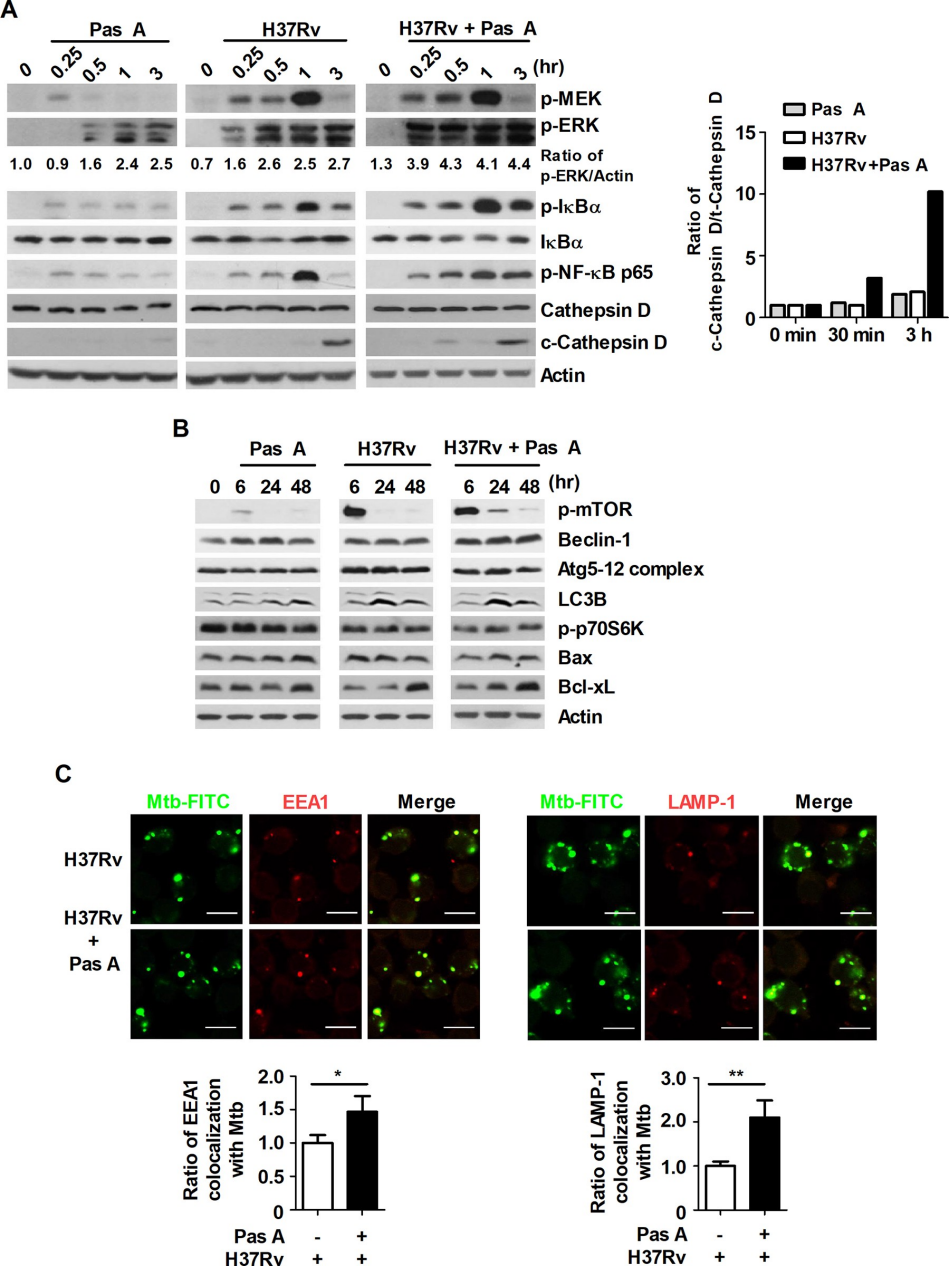
Pasakbumin A activates the NF-κB- and ERK1/2-mediated signaling pathways and induces autophagy in Mtb-infected macrophages. Raw264.7 macrophages were infected with H37Rv (at a MOI of 5), and then treated with pasakbumin A (Pas A, 10 μM) for the indicated time points. Western blot analysis showed the expression for various proteins of (A) the NF-κB, MAPK signaling pathways and (B) the expression of apoptosis- and autophagy-related proteins in pasakbumin A-treated macrophages during H37Rv infection. The band intensity was quantified, and the ratio of p-ERK, LC3-II and cleaved cathepsin D band intensity was shown in bottom of the blot. (C) Raw264.7 cells were infected with FITC-labeled H37Rv, and then treated with pasakbumin A (Pas A, 10 μM) for 3 h. The colocalization of FITC-labeled Mtb and EEA1 or LAMP-1 was monitored by confocal microscopy. Bar graphs represent as fold of change over FITC-labeled Mtb infected cells to indicate the number of FITC-labeled Mtb in EEA1^+^ or LAMP-1^+^ compartment. Bar scale, 20 μM. (A) The full length of all western blots has been provided in the **S2 Fig**. (B) Cropped membrane from different gels was used in western blot assay.

To examine the molecular mechanisms of pasakbumin A during Mtb infection, we investigated the intracellular pathway involved in the pasakbumin A-mediated response to Mtb via activation of various molecules associated with NF-κB and MAPK. The results indicated that the levels of phosphorylated ERK1/2, IκB-α and NF-κB p65 subunit were enhanced in pasakbumin A-treated Raw264.7 cells during Mtb infection compared to cells infected with Mtb alone (**Fig 2A**). Our previous study shown that lysophosphatidylcholine (LPC) can control intracellular Mtb survival by promoting phagosomal maturation in Mtb-infected cells, according to the elevated expression level of cleaved cathepsin D[29]. Cathepsin D is a lysosomal aspartic protease and undergoes several proteolytic processing to produce the mature form at acidic pH. We also investigated that pasakbumin A can control phagosomal maturation in Mtb-infected cells, the expression level of cleaved cathepsin D was detected in Mtb-infected cells. As shown in **Fig 2A**, cathepsin D was rapidly cleaved in pasakbumin-treated cells at 30 min, compared to cells infected Mtb alone. To further investigate whether pasakbumin A enhances phagosome maturation during Mtb infection, we monitored phagosome maturation using FITC-labeled Mtb by their ability to colocalize with early phagosomal marker, EEA1, or late phagosomal marker, LAMP-1. Pasakbumin A promoted the accumulation of EEA1 or LAMP-1 which colocalized with FITC-labeled Mtb (**Fig 2C**).

Our previous report demonstrated that infection with the virulent Mtb strain H37Rv induced more necrosis than the avirulent Mtb strain H37Ra in bone marrow-derived macrophages, leading to host cell death and bacterial dissemination[30]. Therefore, various cell death mechanisms are essential to host defense mechanisms by which host cells remove intracellular pathogens and pathogen-infected host cells[31]. Notably, apoptosis and autophagy play a pivotal role in the pathogenesis as well as in the host defense mechanisms against Mtb infection[32]. We therefore examined whether pasakbumin A affected the induction of different cell death mechanisms, including autophagy and apoptosis. As shown **Fig 2B**, pasakbumin A strikingly increased the phosphorylated mTOR level in Mtb-infected Raw264.7 cells compared to untreated control cells. In addition, the conversion of LC3-I to LC3-II was continued in pasakbumin A-treated cells for 48hr after Mtb infection (**Fig 2B**). Interestingly, pasakbumin A also increased the anti-apoptotic protein Bcl-xL, whereas it decreased the pro-apoptotic protein Bax in H37Rv-infected Raw264.7 cells (**Fig 2B**). These data demonstrated that pasakbumin A induced autophagy with activation of the ERK1/2- and NF-κB signaling pathways in Mtb-infected macrophages. Moreover, pasakbumin A blocked host cell death via apoptosis and enhanced phagosome maturation during Mtb infection.

### Pasakbumin A regulates intracellular Mtb growth and activates the production of inflammatory mediators via the ERK1/2 signaling pathway

**Fig 3:**
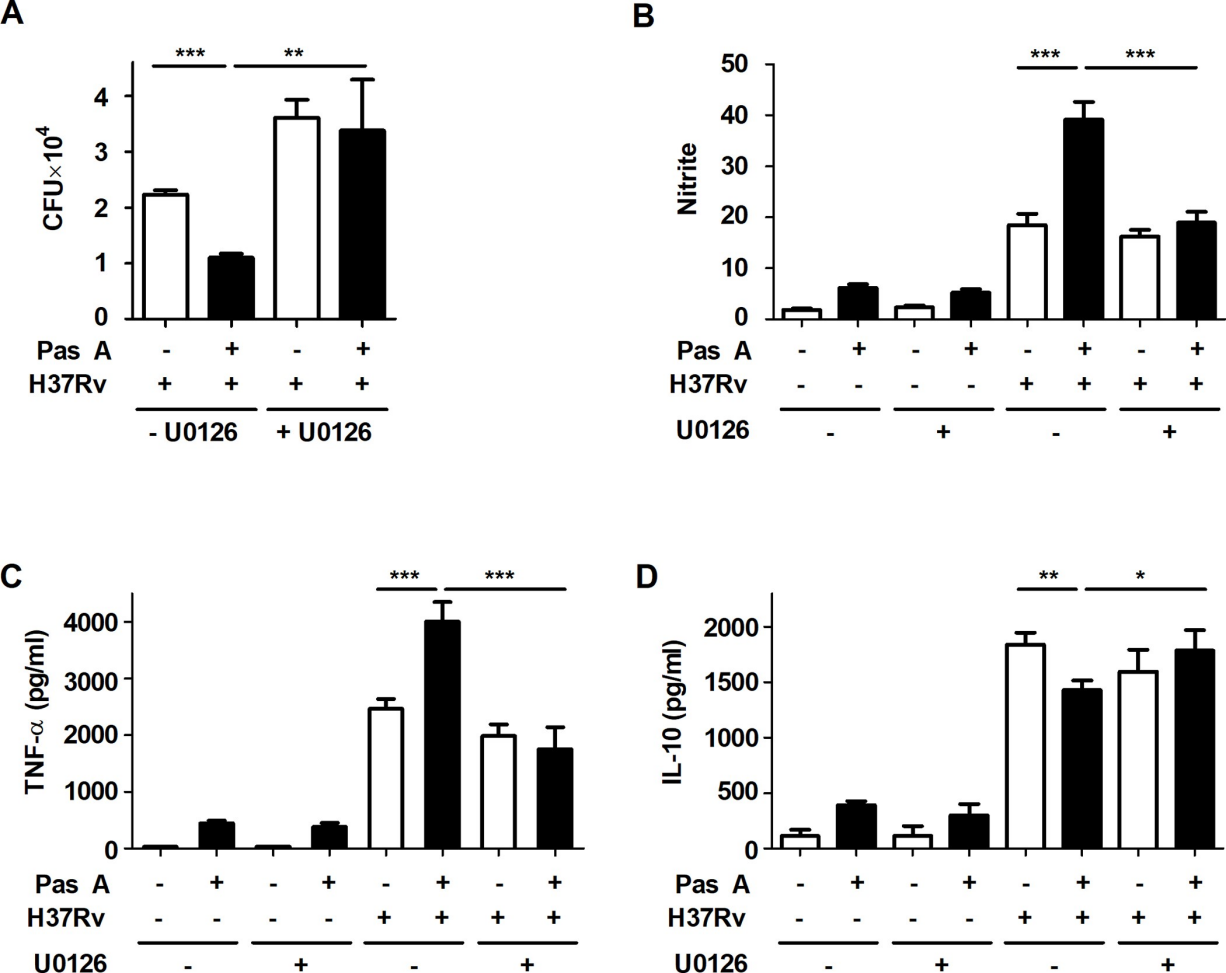
Pasakbumin A controls the intracellular Mtb growth and production of inflammatory mediators through ERK-mediated signaling. Raw264.7 cells were pre-treated with U0126 (10 μM) for 1 h and then stimulated with pasakbumin A (Pas A, 10 μM) during H37Rv infection (MOI of 1 or 5). (A) Intracellular bacterial survival was determined by counting the number of CFUs at 3-weeks after inoculation. (B-D) Culture supernatants were measured for the production of (B) NO, (C) TNF-α and (D) IL-10 at 48 h. The experiments were performed in triplicate. *, *p*<0.05; **, *p*<0.01 and ***, *p*<0.001.

In **Fig 1** we show that pasakbumin A suppressed intracellular Mtb growth and enhanced the production of inflammatory mediators, including TNF-α and NO, in H37Rv-infected Raw264.7 cells. Therefore, to further demonstrate which signaling pathway inhibits intracellular Mtb growth and produces inflammatory mediators in pasakbumin A-treated macrophages during Mtb infection, we focused on the ERK1/2-mediated signaling pathway. We used the pharmacologic inhibitor U0126 to inhibit the ability of MEK1/2 to activate ERK1/2. As shown **Fig 3A**, inhibition of ERK1/2 entirely failed to suppress intracellular Mtb growth in the presence or absence of pasakbumin A during H37Rv infection. In addition, treatment with the ERK1/2 inhibitor decreased NO and TNF-α production, whereas it slightly increased IL-10 production in pasakbumin A-treated Raw264.7 cells during H37Rv infection, similar to the effect observed in cells infected with H37Rv alone (**Fig 3B–3D**). These results indicated that ERK1/2-mediated signaling could control intracellular Mtb growth and to produce inflammatory mediators in pasakbumin A-treated macrophages.

### ERK1/2-mediated signaling induces autophagy in pasakbumin A-treated macrophages during Mtb infection

**Fig 4:**
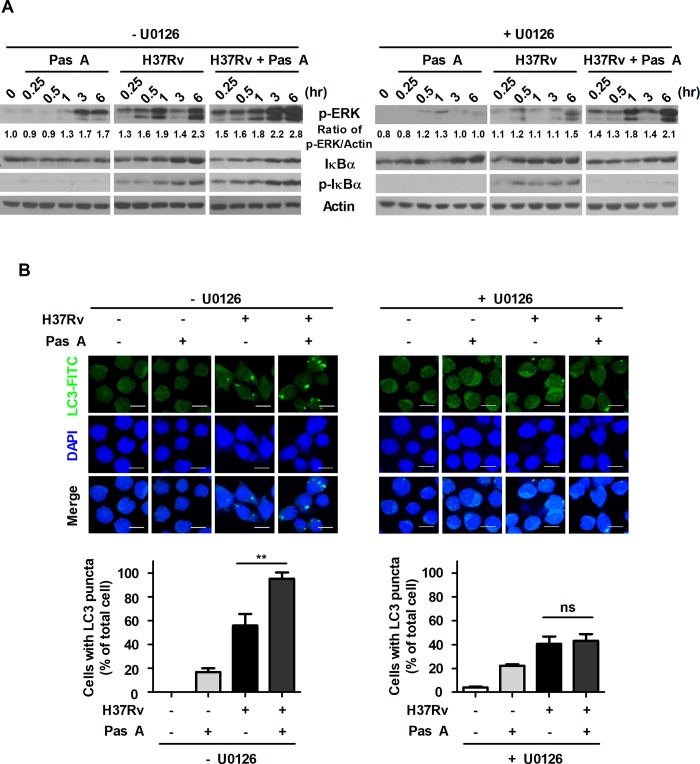
Pasakbumin A induces autophagy by activating ERK1/2-mediated signaling. Raw264.7 cells were pre-treated with U0126 (10 μM) for 1 h and then stimulated with pasakbumin A (Pas A, 10 μM) during H37Rv infection (MOI of 1 or 5). (A) The changes in the phosphorylated and total protein levels of ERK1/2 and IκBα were assessed using western blot analysis. Cropped membrane from different gels was used in western blot assay. The band intensity was quantified, and the ratio of p-ERK band intensity was shown in bottom of the blot. (B) Immunofluorescence staining of LC3 in Mtb-infected cells treated as described above. The number of LC3-positive puncta was counted under a microscope, and the percentage of cells containing LC3-positive puncta relative to the total cell number was calculated. Bar scale, 20 μM. Statistical significance is indicated as *, *p*<0.05 and ns, not significant (*p*>0.05).

A recent study showed that blockade of ERK1/2 inhibits *beclin-1* or *Atg5* mRNA expression in vitamin D3-treated THP-1 cells during Mtb infection, indicating that the induction of autophagy depends on ERK1/2-mediated signaling during Mtb infection[33]. To assess whether pasakbumin A-induced autophagy is triggered by ERK1/2-mediated signaling in Mtb-infected macrophages, we examined the induction of autophagy in pasakbumin A-treated Raw264.7 cells in the presence or absence of U0126. Treatment with U0126 diminished the phosphorylated level of ERK1/2 and IκBα in pasakbumin A-treated Raw264.7 cells during H37Rv infection, similar to those found in Mtb-infected Raw264.7 cells without U0126 (**Fig 4A**). Treatment with pasakbumin A enhanced the number of endogenous LC3-positive puncta, which indicated autophagosomes, in Mtb-infected Raw264.7 cells compared with those infected with H37Rv without pasakbumin A (**Fig 4B**). However, the number of endogenous LC3-positive puncta was significantly reduced by the ERK1/2 inhibitor U0126 in pasakbumin A-treated Raw264.7 cells during H37Rv infection, similar to those infected with H37Rv alone (**Fig 4B**). These results indicated that ERK1/2-mediated signaling was important for autophagy induction after pasakbumin A treatment.

### Combined treatment with an anti-TB drug improves mycobactericidal activity of pasakbumin A in Mtb-infected macrophages

**Fig 5:**
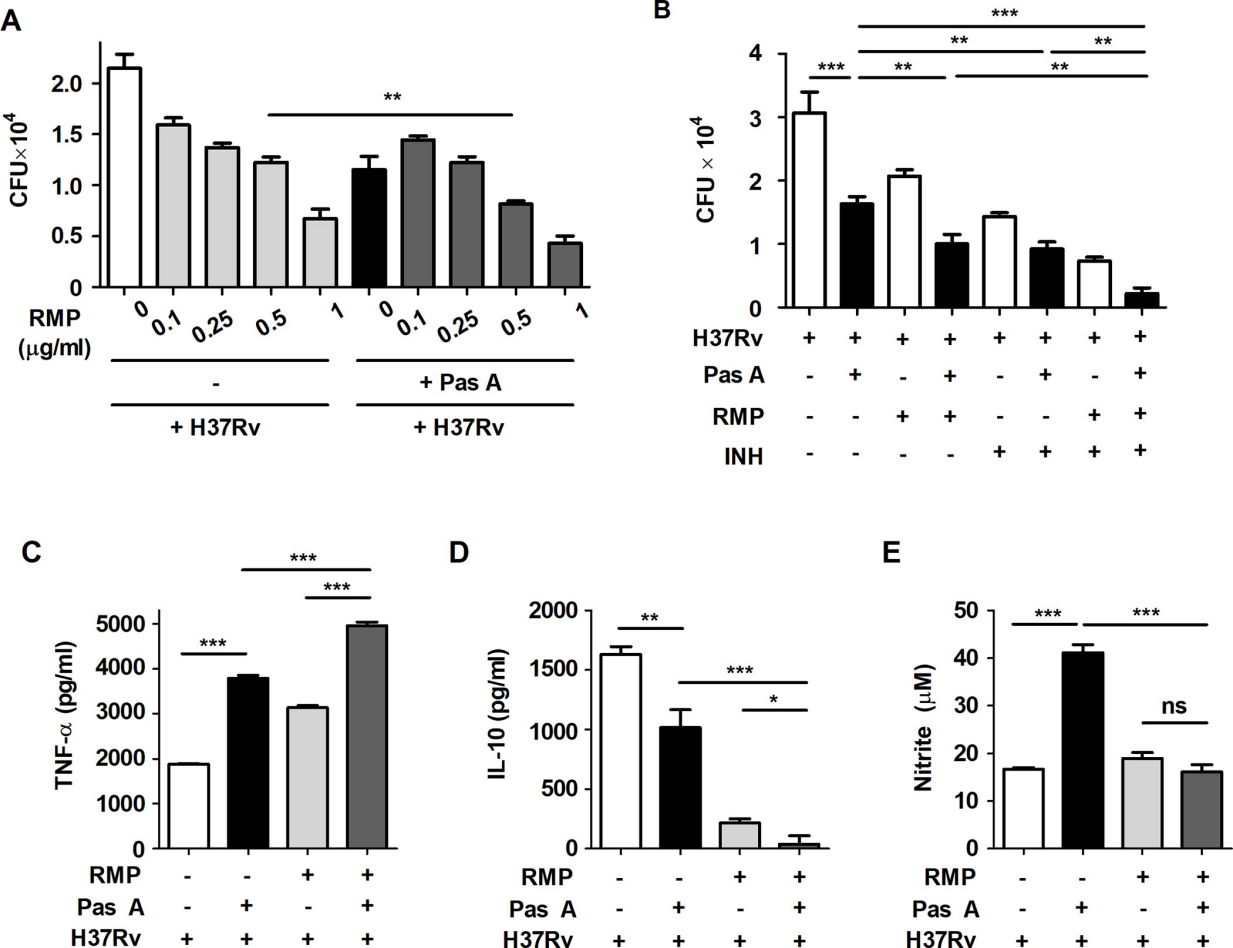
Combination treatment with pasakbumin A and an anti-TB drug improves the antibacterial immunity in Mtb-infected macrophages. (A) Raw264.7 macrophages were infected with H37Rv (at MOI of 1 or 5) for 4 h and treated with pasakbumin A (Pas A, 10 μM) alone or Pas A combined with an anti-TB drug, rifampicin (RMP), for 48 h in a dose-dependent manner. Intracellular bacterial survival was determined by counting the number of CFUs at 3-weeks after inoculation. (B) Raw264.7 macrophages were infected with H37Rv for 4 h and treated with Pas A (10 μM) alone, Pas A and RMP (0.5 μg/ml), Pas A and isoniazid (INH, 0.1 μg/ml) or Pas A and RMP/INH for 48 h. Intracellular bacterial survival was determined by counting the number of CFUs. (C, D) Culture supernatants were measured for the production of (C) TNF-α and (D) IL-10 with ELISA at 48 h. (E) NO production was detected in cell culture supernatants. Statistical significance is indicated as *, *p*<0.05; **, *p*<0.01, ***, *p*<0.001 and ns, not significant (*p*<0.05).

Recently, a published report showed that treatment with the anti-TB drugs INH and RMP increased cellular and mitochondrial ROS, leading to autophagy activation in Mtb-infected macrophages. These findings indicated that antibiotics-induced autophagy plays an important role in chemotherapy against Mtb infection[34]. We next questioned whether combined treatment with an anti-TB drug is more efficacious than treatment with pasakbumin A alone for infection with Mtb. To investigate this, we assessed intracellular bacterial growth in Mtb-infected Raw264.7 cells in the presence or absence of an anti-TB drug RMP. As shown **Fig 5A**, treatment with RMP suppressed intracellular bacterial growth in a dose-dependent manner. Notably, RMP at a dose with significant antimicrobial activities (0.5 μg/ml for RMP) significantly inhibited intracellular bacterial survival in pasakbumin A-treated cells during Mtb infection compared to that of Mtb-infected cells (**Fig 5A**). To further demonstrate whether the combined treatment with an anti-TB drug more enhances the antimycobactericidal effect of pasakbumin A, we investigated intracellular Mtb growth using other anti-TB drug, isoniazid (INH). Similar to RMP, the combined treatment of INH and pasakbumin A significantly reduced intracellular Mtb growth (**Fig 5B**). In addition, intracellular Mtb growth was nearly decreased in cells treated with pasakbumin A, RMP and INH (**Fig 5B**). TNF-α secretion was irregularly increased by RMP in a dose-dependent manner; however, more was produced in Mtb-infected cells treated with pasakbumin A and RMP than Mtb-infected cells treated with RMP alone (**Fig 5C**). In contrast, combined treatment of pasakbumin A and RMP showed stronger decreases in IL-10 secretion than that of cells treated with pasakbumin A in the absence of RMP during Mtb infection (**Fig 5D**). There was no significant difference in NO production after RMP treatment in pasakbumin A-treated cells (**Fig 5E**). These data suggested that the combination of pasakbumin A with an anti-TB drug effectively suppressed intracellular Mtb growth by promoting the production of pro-inflammatory cytokine and blocking the production of anti-inflammatory cytokine in macrophages

### RMP strongly accelerates pasakbumin A-induced autophagy through ERK1/2-mediated signaling in Mtb-infected macrophages

**Fig 6:**
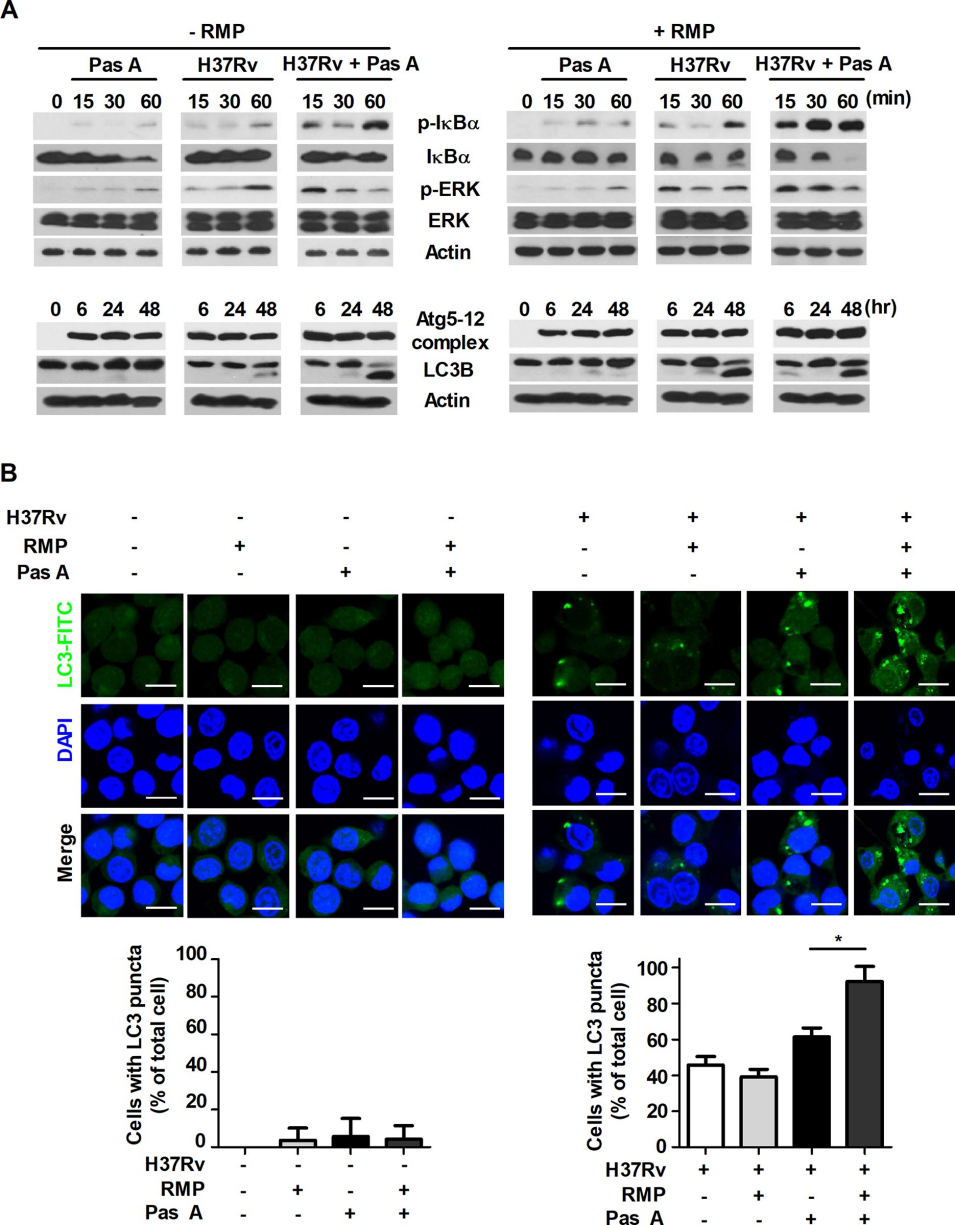
Treatment of pasakbumin A combined with RMP accelerates autophagy and the activation of NF-κB- and ERK1/2-mediated signaling compared to that of cells treated with pasakbumin A alone during Mtb infection. (A) Raw264.7 macrophages were infected with H37Rv (at a MOI of 5), and then treated with pasakbumin A (Pas A, 10 μM) in presence or absence of RMP for the indicated time points. (A) The expression levels of autophagy-related proteins as well as phosphorylated and total protein for various components of the NF-κB and MAPK signaling pathways were examined by western blot assay. Cropped membrane from different gels was used in western blot assay. (B) Raw264.7 macrophages were infected with H37Rv (at a MOI of 5) for 4 h, and then treated with pasakbumin A (Pas A, 10 μM) in presence or absence of RMP for 48 h. The LC3-positive macrophages after treatment with Pas A with or without RMP during Mtb infection were detected via immunofluorescence staining. The number of LC3-positive puncta was counted under a microscope, and the percentage of cells containing LC3-positive puncta relative to the total cell number was calculated. Bar scale, 20 μM. Statistical significance is indicated as *, *p*<0.05.

Our present data showed that pasakbumin A induced autophagy via ERK1/2-mediated signaling, and combined treatment with RMP substantially reduced intracellular bacterial growth in Mtb-infected macrophages. Thus, we investigated the cellular mechanism by which combined treatment of pasakbumin A and RMP enhances autophagy to control intracellular Mtb growth in macrophages. Phosphorylated levels of ERK1/2 and IκBα rapidly increased after the combined treatment with pasakbumin A and RMP in Mtb-infected cells compared with those treated with pasakbumin A alone (**Fig 6A**). We also found that combined treatment of pasakbumin A and RMP strongly induced the conversion of LC3-I to LC3-II in Mtb-infected cells (**S3D Fig and Fig 6A**). To further confirm this observation, we detected endogenous LC3 by immunofluorescence staining. After combined treatment of pasakbumin A and RMP, the percentage of FITC-LC3-positive cells with punctate staining was greater than that of Mtb-infected cells treated with pasakbumin A alone (**Fig 6B**).

**Fig 7:**
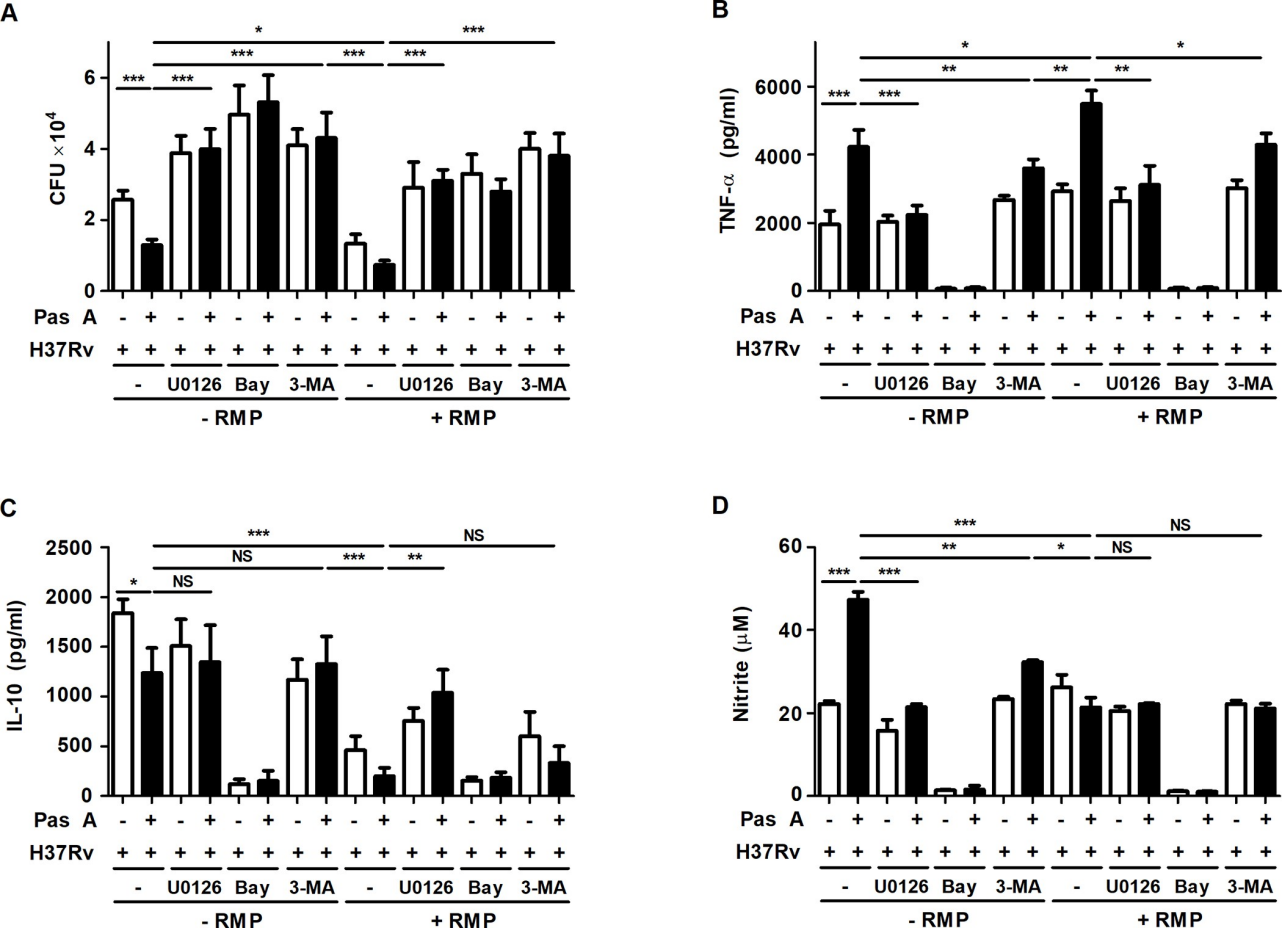
Treatment of pasakbumin A combined with RMP effectively controls intracellular Mtb growth by accelerating autophagy via NF-κB- and ERK1/2-mediated signaling. Raw264.7 macrophages were pre-treated with ERK1/2 inhibitor (U0126, 10 μM), NF-κB inhibitor (bay 11–7082, 10 μM) or autophagy inhibitor (3-MA, 5 mM) for 1 h and infected with H37Rv for 4 h. After 4 h, cells were treated with pasakbumin A (Pas A, 10 μM) in presence or absence of RMP for 48 h. (A) Intracellular bacterial survival was determined by counting the number of CFUs at 3-weeks after inoculation. (B, C) Culture supernatants were measured for the production of (B) TNF-α and (C) IL-10 with ELISA at 48 h. (D) NO production was detected in cell culture supernatants. Statistical significance is indicated as *, *p*<0.05; **, *p*<0.01, ***, *p*<0.001 and ns, not significant (*p*<0.05).

To ensure that the combined treatment of pasakbumin A and anti-TB drug effectively controls intracellular Mtb growth by inducing autophagy through ERK1/2- and NF-κB-mediated signaling pathway, we investigated intracellular Mtb growth using each inhibitors to block these signaling in presence or absence of anti-TB drug, RMP. Intracellular Mtb growth was more reduced in cells treated with pasakbumin A and RMP than in cells treated with pasakbumin A alone or RMP alone. However, blockade of ERK1/2 using U0126 or IκBα using Bay 11–7082 restored intracellular Mtb growth in pasakbumin A-treated cells in presence of RMP during Mtb infection (**Fig 7A**). Pre-treatment of 3-MA was also recovered intracellular Mtb growth in pasakbumin A-treated cells (**Fig 7A**). In addition, pre-treatment of U0126 or Bay11-7082 reduced the production of TNF-α and NO in pasakbumin A-treated cells during Mtb infection (**Fig 7B and 7D**). Production of IL-10 was not different in presence or absence of pasakbumin A when cells were pre-treated each inhibitors (**Fig 7C**). These data suggest that pasakbumin A may be an effective adjuvant for enhancing the host-directed immunotherapy against tuberculosis.

## Discussion

TB, which is caused by Mtb, remains a major infectious disease despite the advances made in treatment and management[1]. TB can be effectively treated with a multidrug regimen of first-line drugs, including INH, RMP, PZA, EMB and streptomycin (SM), for at least 6 months[35]. However, first-line therapy often fails to cure TB for several reasons, including patient non-compliance, inappropriate drug levels, drug shortages or a number of other factors[36]. In this context, Menzies *et al* showed that TB patients have failed to treat TB because they does not take anti-TB drugs correctly, according to the Montreal Chest Hospital in 1987–1988[37].

The multidrug regimen of anti-TB drugs has also been associated with an increased incidence of adverse effects that cause mild to severe host damage; these effects may cause discontinuation of the treatment due to the poor health and immunity of TB patients[20]. Common adverse effects are dizziness, muscular twitching, loss of vision or hearing, hemolytic anemia, acute renal failure, hepatotoxicity and thrombocytopenia[38, 39]. Recently, Yakar *et al* showed that thrombocytopenia is induced by INH and RMP, and patients had no further thrombocytopenia development without INH and RMP during the therapeutic period[40]. Furthermore, many other studies have demonstrated the regulatory effects of RMP on the immune response, including phagocytosis, antibody production, T cell differentiation and delayed hypersensitivity[41]. Due to these problems, there is an urgent need to identify novel anti-TB agents.

*E*. *longifolia* (known as tongkat ali) is a commonly distributed and popular traditional herbal medicine in Southeast Asia and Indo-China used to treat various illnesses, including sexual dysfunction, fever, malaria, ulcers, high blood pressure, TB, and diarrhea[26]. The plant parts of *E*. *longifolia* have a variety bioactive compounds, such as quassinoids, alkaloids and squalene derivatives[42]. Tada *et al*. isolated four quassinoids, pasakbumin-A, -B, -C, and–D, from the roots and showed that pasakbumin-A and -B have anti-ulcer activity[26]. Many studies have also shown that pasakbumin-A, -B, -C, and -D have strong cytotoxicity toward human lung cancer (A549) and human breast cancer (MCF-7) cell lines[43, 44]. Farouk and Benafri demonstrated that the aqueous extracts of leaves exhibit antibacterial activity against *Staphylococcus aureus* and *Serratia marscesens*[27], however, antibacterial activity against other pathogenic microorganisms has not been determined. In the present study, pasakbumin A extracted from *E*. *longifolia* was shown to have anti-TB activity against the virulent Mtb strain H37Rv in Raw264.7 macrophage cell line. As a result, our hypothesis of the anti-TB activity of pasakbumin A appears to be accurate because pasakbumin A inhibited intracellular Mtb growth in mouse macrophages. However, pasakbumin A showed little cytotoxic activity in the present study on Mtb and mouse macrophages. In contrast, in H37Rv-infected macrophages, pasakbumin A protected the host cells from Mtb-induced apoptotic cell death. Our results suggest that pasakbumin A can be developed as a safe and continuously available therapeutic drug to protect hosts from cell death and interfere with the growth of intracellular Mtb.

Autophagy is a lysosomal self-degradation process for cellular homeostasis and functions as an innate defense mechanism during Mtb infection. Gutierrez *et al*. first showed that induction of autophagy by starvation or treatment with the mTOR inhibitor rapamycin increases co-localization of Mtb with LC3 and beclin-1 and delivers Mtb to phagolysosomes, suggesting that autophagy plays an important role as a defense mechanism against TB[45]. Our results also suggested that activation of autophagy induced by pasakbumin A may play a central role in its antibacterial effects.

Many studies have demonstrated that several antibiotics can induce or suppress autophagy. Kim *et al*. showed that INH and PZA promoted autophagy activation and phagosomal maturation in Mtb-infected host cells, suggesting that host autophagy plays an important role in host protective responses during antibiotic chemotherapy against TB[34]. In this context, we observed that the combined treatment of pasakbumin A and the anti-TB drug RMP resulted in increased intracellular LC3 distribution and phosphorylated ERK1/2 and IκBα in Mtb-infected cells compared to cells infected Mtb alone. These results reveal that the combined treatment of pasakbumin A with an anti-TB drug resulted in effective antimicrobial activity against Mtb infection by inducing autophagy and that pasakbumin A also protects host cells from apoptotic cell death during Mtb infection.

In conclusion, we report that pasakbumin A inhibited intracellular Mtb growth in mouse macrophages by activating autophagy through the ERK1/2-mediated signaling pathway. This compound also increased the levels of NO and pro-inflammatory cytokine via the ERK1/2- and NF-κB-mediated signaling pathways in Mtb-infected cells but not the anti-inflammatory cytokine IL-10. Treatment with the anti-TB drug RMP in combination with pasakbumin A significantly suppressed intracellular Mtb growth by promoting autophagy and enhancing TNF-α production via the ERK1/2-mediated signaling pathway. Taken together, understanding the molecular mechanisms of pasakbumin A during Mtb infection will provide insights into the development of novel therapeutic anti-TB drugs and potential HDT strategies that modulate the host immune response against Mtb infection.

## Materials and methods

### Cell culture and Mtb infection

The mouse macrophage cell line, Raw264.7, was purchased from ATCC (ATCC, Rockville, MD, USA). Cells were maintained in DMEM (Cellgro, Herndon, VA, USA) supplemented with 10% fetal bovine serum (FBS, Atlas Biologicals, Fort Collins, CO) and 1% penicillin/streptomycin (Corning Incorporated, Corning, NY, USA). The human monocytic cell line, THP-1, was purchased from Korean Cell Line Bank (Korean Cell Line Bank, Korea) and maintained RPMI-1640 (Cellgro, Herndon, VA, USA) supplemented with 10% FBS and 1% penicillin/streptomycin. THP-l cells were treated with 5 ng/ml of phorbol myristate acetate (PMA, Sigma-Aldrich, St. Louis, MO, USA) for 24 h to induce differentiation into macrophages. Cells were cultured in a standard cell culture incubator at 37 °C with an atmosphere of 5% CO_2_ and 95% air. For Mtb infection, cells were seeded at density of 2ⅹ10^4^/well and infected with Mtb for 4 h at MOI of 1 or 5. After 4 h, cells were washed with PBS twice to remove extracellular bacteria and cultured in complete medium without penicillin/streptomycin.

### Reagent

Pasakbumin A was isolated from the roots of *E*. *longifolia* as previously described[44]. Rifampicin (RMP) and isoniazid (isonicotinic acid hydrazide, INH) were purchased from Tokyo Chemical Industry Co. Ltd. (TCI Co. Ltd., Tokyo, Japan). Other reagents were as follows: U0126 (Cell Signaling, Danvers, MA, USA), 3-methyladenine (3-MA, Sigma-Aldrich, St. Louis, MO, USA), and Bay11-7082 (Cayman Chemical, MI, USA).

### Bacterial strains and culture conditions

*M*. *tuberculosis* strain H37Rv was used in this study. Mtb was grown in Middlebrook 7H9 broth (Difco Laboratories, USA) supplemented with 10% ADC (5% bovine albumin, 2% dextrose, 0.03% catalase, 0.85% sodium chloride) and 0.2% glycerol at 37°C. After 3 weeks of culture, Mtb was harvested, adjusted to 1ⅹ10^7^ bacteria/200 μl stock solution, aliquoted, and maintained at -70°C until used.

### Enzyme-linked immunosorbent assay (ELISA)

Cultured supernatants were collected and tested for TNF-α and IL-10 production using an ELISA kit (Peprotech, NJ, USA) according to the manufacturer's instructions. Samples were read at 450 nm using a microplate reader (Biotek Instruments Inc., Winooski, VT, USA).

### Nitric oxide (NO) detection assay

NO production was measured using a nitric oxide detection kit (Intron Bio-technology Inc., Kyungki-Do, Korea) according to the manufacturer’s procedure. NO detection assay was performed as previously described[29]. Briefly, culture supernatants were collected and mixed with N1 buffer (sulfanilamide in the reaction buffer) for 10 min at room temperature. After 10 min, the mixture was combined with N2 buffer (naphthylethylenediamine in the stabilizer buffer) for 10 min at room temperature, and absorbance was measured between 520–560 nm using a plate reader. NO production was calculated from a standard curve with nitrite standard solution.

### Western blot assay

For cell lysates, cells were lysed with lysis buffer containing complete a protease inhibitor cocktail (Calbiochem, San Diego, CA, USA). Western blotting was performed as previously described[46]. The acrylamide percentage in SDS-PAGE gel was determined by the size of the target protein in the samples. The membranes were stripped or cropped to detect various proteins. Anti-NOS2, anti-IκBα, anti-NF-κB p65, anti-Cathepsin D, anti-Bax, anti-Bcl-xL and anti-β-actin were purchased from Santa Cruz Biotechnology (Santa Cruz, CA, USA). Anti-phospho-MEK1/2, anti-phospho-ERK1/2, anti-phospho-IκBα, anti-phospho-mTOR, anti-beclin-1, anti-Atg5-12 complex, anti-LC3B and anti-phospho-p70S6K were purchased from Cell Signaling (Danvers, MA, USA). The band intensity was quantified using Image J program, and presented in bottom of the blot.

### Colony-forming unit (CFU) assay

Mtb-infected macrophages were lysed with 0.1% saponin (Sigma-Aldrich, Dorset, UK) for 10 min at 37°C with 5% CO_2_ and then serially diluted in Middlebrook 7H9 broth. After then, samples were plated on Middlebrook 7H10 agar in triplicate and incubated at 37°C for 21 days. CFUs were counted at 21 days after incubation.

### Immunofluorescence analysis

Immunofluorescence analysis was performed as previously described[47]. Briefly, cells were seeded onto cover-slides and treated as described above. Cells were washed in PBS, fixed in 4% paraformaldehyde overnight, and permeabilized in 0.1% Triton X-100 for 15 min. Slides were incubated with the primary antibodies (anti-LC3B, MBL, Nagoya, Japan) at room temperature for 2 h and washed 3 times with PBS for 5 min. Then, the slides were incubated with FITC anti-rabbit secondary antibody (Jackson Immunoresearch, West Grove, USA) at room temperature for 1 h 30 min, and nuclei were counterstained with 4'-6-diamidino-2-phenylindole (DAPI, Sigma-Aldrich, St. Louis, USA) for 5 min. For the quantification of LC3-positive puncta, LC3-positive cells were selected and counted about 30 to 50 cells in images. Data were expressed as the percentage of LC3-positive puncta over the number of total cells.

For the detection of phagosome maturation, cells were infected with FITC-labeled Mtb for 4 h and then treated with pasakbumin A for 3 h. Cells were washed in PBS, fixed and permeabilized. Slides were stained with Alexa Fluor 647 anti-mouse CD107a LAMP-1 (BioLegend, San Diego, CA, USA) or anti-EEA1-Alexa Fluor 647 (MBL, Woburn, MA, USA) for 2 h at room temperature. Quantification of the colocalization of Mtb and each phagosomal marker were previously described[29]. Cover-slides were mounted in Fluoromount-G and examined by confocal microscopy (FV1000 SPD, Olympus, Tokyo, Japan).

### Cell viability assay

Raw264.7 cells were infected with H37Rv at a MOI of 1 for 4 h and then treated with pasakbumin A for 72 h. Cells were suspended and stained with trypan blue solution (Sigma-Aldrich, MO, USA), which selectively stains dead cells. The cell number was determined by a hemocytometer.

### Statistical analysis

The results were presented as the mean±SD of triplicate experiments. Statistical significance was analyzed using Student’s t-test and one-way ANOVA followed by Tukey's post hoc test for multiple comparisons. Data were graphed and analyzed using GraphPad Prism software (GraphPad Software, La Jolla, CA, USA). Values of p<0.05 were considered to be significant. Statistical significance is indicated as *, *p*<0.05; **, *p*<0.01; ***, *p*<0.001; and ns, not significant (*p*>0.05).

## Supporting information

S1 Fig Full scans of Western blots. (PDF)

S2 Fig Full scans of Western blots. (PDF)

S3 Fig Treatment of pasakbumin A promotes autophagy and prevents host cell death during Mtb infection. (A) Raw 264.7 cells were infected with H37Rv for 4 h, and then treated with pasakbumin A for 48 h. Cells were stained with annexin-V/PI to screen the infection of cell death and analyzed by flow cytometry. Bar graphs represent the percentage of annexin V^-^/PI^+^ cells (top panel) or annexin V^+^/PI^+^ cells (bottom panel). (B) Cell death was also determined by LDH release. (C) PMA-differentiated THP-1 cells were infected with H37Rv for 4 h, and then treated with pasakbumin A for 48 h. Intracellular bacterial survival was determined by counting the number of CFUs at 3-weeks after inoculation. (D) Raw 264.7 cells were pre-treated with bafilomycin A1 (Baf A1, 1 μM) for 2 h, and then infected with H37Rv for 4 h. After 4 h, cells were treated with pasakbumin A for 6 h in presence or absence of RMP. The conversion of LC3-I to LC3-II was detected using western blot assay. The band intensity was quantified, and the ratio of LC3-II band was shown in the bottom of panel. Statistical significance is indicated as *, *p*<0.05; **, *p*<0.01 and ns, not significant (*p*<0.05).
(PDF)

S1 Table Minimal data set in this study. (PDF)
