# Supplementary material for: Pasakbumin A controls the growth of Mycobacterium tuberculosis by enhancing the autophagy and production of antibacterial mediators in mouse macrophages
Source: PLoS One. 2019 Mar 13;14(3):e0199799. doi: 10.1371/journal.pone.0199799 (PMC6415846; doi:10.1371/journal.pone.0199799)
Supplement: S2 Fig — (PDF) [file pone.0199799.s002.pdf]

Supplementary information

Pasakbumin A controls the growth of *Mycobacterium tuberculosis* by enhancing the autophagy and production of antibacterial mediators in mouse macrophages

Hyo-Ji Lee[1], Hyun-Jeong Ko[2], Seung Hyun Kim[3] and Yu-Jin Jung[1*]

[1] Department of Biological Sciences and Institute of Life Sciences, Kangwon National University, Chuncheon, 24341, Republic of Korea

[2] College of Pharmacy, Kangwon National University, Chuncheon, 24341, Republic of Korea

[3] College of Pharmacy, Yonsei University, Incheon, 21983, Republic of Korea

*Corresponding author: Yu-Jin Jung, Department of Biological Sciences and Institute of Life Sciences, Kangwon National University, Chuncheon, 24341, Republic of Korea. Tel: +82-33-250-8533; Fax: +82-33-251-3990 ; E-mail: yjjung@kangwon.ac.kr

Pas A	H37Rv	H37Rv+PasA

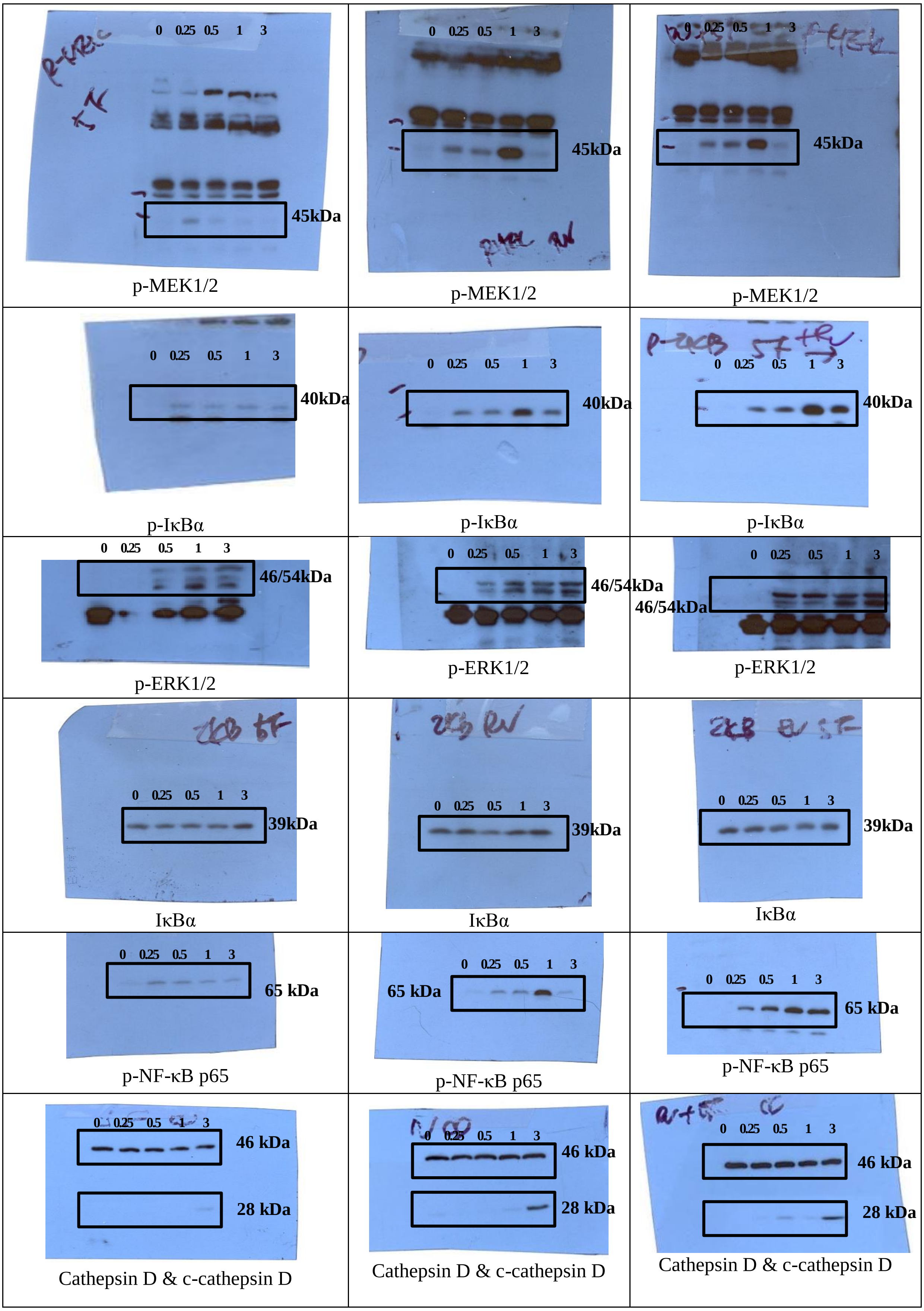

Figure 2A
