# Supplementary material for: Pasakbumin A controls the growth of Mycobacterium tuberculosis by enhancing the autophagy and production of antibacterial mediators in mouse macrophages
Source: PLoS One. 2019 Mar 13;14(3):e0199799. doi: 10.1371/journal.pone.0199799 (PMC6415846; doi:10.1371/journal.pone.0199799)
Supplement: S3 Fig — (A) Raw 264.7 cells were infected with H37Rv for 4 h, and then treated with pasakbumin A for 48 h. Cells were stained with annexin-V/PI to screen the infection of cell death and analyzed by flow cytometry. Bar graphs represent the percentage of annexin V-/PI+ cells (top panel) or annexin V+/PI+ cells (bottom panel). (B) Cell death was also determined by LDH release. (C) PMA-differentiated THP-1 cells were infected with H37Rv for 4 h, and then treated with pasakbumin A for 48 h. Intracellular bacterial survival was determined by counting the number of CFUs at 3-weeks after inoculation. (D) Raw 264.7 cells were pre-treated with bafilomycin A1 (Baf A1, 1 μM) for 2 h, and then infected with H37Rv for 4 h. After 4 h, cells were treated with pasakbumin A for 6 h in presence or absence of RMP. The conversion of LC3-I to LC3-II was detected using western blot assay. The band intensity was quantified, and the ratio of LC3-II band was shown in the bottom of panel. Statistical significance is indicated as *, p<0.05; **, p<0.01 and ns, not significant (p<0.05). (PDF) [file pone.0199799.s003.PDF]

Supplementary information

Pasakbumin A controls the growth of *Mycobacterium tuberculosis* by enhancing the autophagy and production of antibacterial mediators in mouse macrophages

Hyo-Ji Lee[1], Hyun-Jeong Ko[2], Seung Hyun Kim[3] and Yu-Jin Jung[1*]

[1] Department of Biological Sciences and Institute of Life Sciences, Kangwon National University, Chuncheon, 24341, Republic of Korea

[2] College of Pharmacy, Kangwon National University, Chuncheon, 24341, Republic of Korea

[3] College of Pharmacy, Yonsei University, Incheon, 21983, Republic of Korea

*Corresponding author: Yu-Jin Jung, Department of Biological Sciences and Institute of Life Sciences, Kangwon National University, Chuncheon, 24341, Republic of Korea. Tel: +82-33-250-8533; Fax: +82-33-251-3990 ; E-mail: yjjung@kangwon.ac.kr

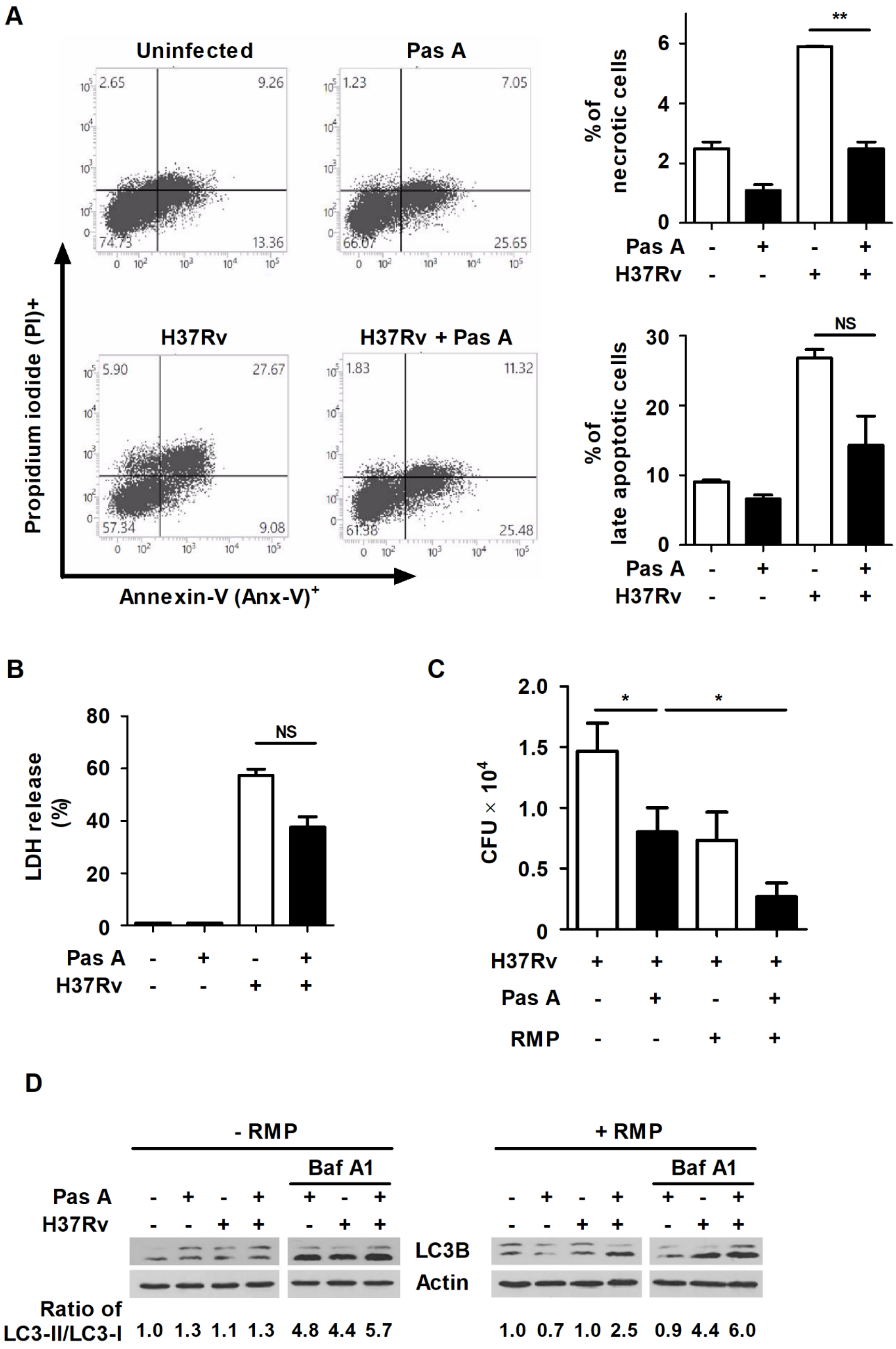
A
Uninfected
Pas A
H37Rv
H37Rv + Pas A
2.65
9.26
74.73
13.36
1.23
7.05
66.07
25.65
5.90
27.67
57.34
9.08
1.83
11.32
61.38
25.48
Propidium iodide (PI)+
Annexin-V (Anx-V)+
% of necrotic cells
**
% of late apoptotic cells
NS
Pas A - + - +
H37Rv - - + +
B
LDH release (%)
NS
Pas A - + - +
H37Rv - - + +
C
CFU × 10^4
*
*
H37Rv + + + +
Pas A - + - +
RMP - - + +
D
- RMP
+ RMP
Baf A1
Baf A1
Pas A - + - + + - +
H37Rv - - + + - + +
LC3B
Actin
Ratio of LC3-II/LC3-I
1.0 1.3 1.1 1.3 4.8 4.4 5.7
1.0 0.7 1.0 2.5 0.9 4.4 6.0
