# Supplementary material for: Pasakbumin A controls the growth of Mycobacterium tuberculosis by enhancing the autophagy and production of antibacterial mediators in mouse macrophages
Source: PLoS One. 2019 Mar 13;14(3):e0199799. doi: 10.1371/journal.pone.0199799 (PMC6415846; doi:10.1371/journal.pone.0199799)
Supplement: S1 Table — (PDF) [file pone.0199799.s004.pdf]

Figure A1

	CFU		
	**Mean**	**SD**	**N**
H37Rv	2.05	0.07924645	6
H37Rv+Pas A	1.116667	0.07420692	6

Figure B1

	OD600		
	**Mean**	**SD**	**N**
H37Rv	0.4965	0.01343503	2
H37Rv+Pas A	0.4885	0.0106066	2

	CFU		
	**Mean**	**SD**	**N**
H37Rv	1.75	0.212132	2
H37Rv+Pas A	1.833333	0.09428091	2

Figure C1

	Cell number		
	**Mean**	**SD**	**N**
Non-infection	25.73333	0.8326664	3
Pas A	23.86667	0.1973153	3
H37Rv	2.573333	0.1154701	3
H37Rv+Pas A	4.093333	0.1006645	3

Figure D1

	TNF		
	**Mean**	**SD**	**N**
Non-infection	27.58667	4	3
Pas A	445.2	42.77071	3
H37Rv	2266.833	177.5528	3
H37Rv+Pas A	3915.167	46.18802	3

	IL-10		
	**Mean**	**SD**	**N**
Non-infection	132.2917	45.06939	3
Pas A	332.2917	21.65063	3
H37Rv	1838.667	109.6965	3
H37Rv+Pas A	1428.667	87.36895	3

Figure E1

	NO		
	Mean	SD	N
Non-infection	2.085859	0.3942549	3
Pas A	7.588384	0.5467332	3
H37Rv	20.22727	1.893939	3
H37Rv+Pas A	42.94192	1.44652	3

Figure C2

	EEA1		
	Mean	SD	N
H37Rv	1	0.11845	3
H37Rv+PasA	1.46618	0.23441	3

	LAMP-1		
	Mean	SD	N
H37Rv	1	0.101096	3
H37Rv+PasA	2.094438	0.392422	3

Figure A3

	CFU		
	Mean	SD	N
H37Rv	2.23	0.0792465	3
Pas A	1.09867	0.0742069	3
U0126+H37Rv	3.61	0.32563	3
U0126+Pas A	3.38	0.912853	3

Figure B3

	NO		
	Mean	SD	N
Non-infection	1.85859	0.2493549	3
Pas A	6.112434	0.7354332	3
U0126	2.31589	0.33198	3
U0126+Pas A	5.23345	0.64935	3
H37Rv	18.45227	2.193939	3
H7Rv+PasA	39.14192	3.44652	3
U0126+H37Rv	16.23345	1.24635	3
U0126+H37Rv+PasA	18.95832	2.123333	3

Figure C3

	TNF		
	Mean	SD	N
Non-infection	27.58667	4	3
Pas A	445.2	42.77071	3
U0126	32.19753	2.21333	3
U0126+Pas A	381.2612	69.11465	3
H37Rv	2465.664	177.5528	3
H7Rv+PasA	4002.199	346.188	3
U0126+H37Rv	1986.553	201.2235	3
U0126+H37Rv+PasA	1750.327	388.2166	3

Figure D3

	IL-10		
	Mean	SD	N
Non-infection	112.2917	55.16939	3
Pas A	392.8917	33.65063	3
U0126	113.7192	88.21633	3
U0126+Pas A	299.7717	101.2202	3
H37Rv	1838.667	109.6965	3
H7Rv+PasA	1428.667	87.36895	3
U0126+H37Rv	1592.78	202.6567	3
U0126+H37Rv+PasA	1787.2	181.3394	3

Figure B4

	-U0126 LC3 dot		
	Mean	SD	N
Non-infection	0	0	3
Pas A	16.7183	3.2264	3
H37Rv	55.91862	9.614235	3
H37Rv+Pas A	95.22334	5.12469	3

	+U0126 LC3 dot		
	Mean	SD	N
U0126+Non-infecion	4.012433	0.62541	3
U0126+Pas A	22.22333	1.115003	3
U0126+H37Rv	40.55555	6.250433	3
U0126+H37Rv+Pas A	43.11624	5.78331	3

Figure A5

	CFU		
	Mean	SD	N
H37Rv	2.15	0.1352775	3
H37Rv+RMP 0.1	1.59	0.07211103	3
H37Rv+RMP 0.25	1.37	0.04358899	3
H37Rv+RMP 0.5	1.226667	0.05033223	3
H37Rv+RMP 1	0.6733333	0.09291573	3
H37Rv+PasA	1.153333	0.1301281	3
H37Rv+PasA+RMP 0.1	1.446667	0.03511884	3
H37Rv+PasA+RMP 0.25	1.223333	0.05859465	3
H37Rv+PasA+RMP 0.5	0.8166667	0.0305505	3
H37Rv+PasA+RMP 1	0.43	0.07211103	3

Figure B5

	CFU		
	Mean	SD	N
H37Rv	3.066667	0.332916	3
H37Rv+PasA	1.633333	0.11547	3
H37Rv+RMP	2.066667	0.104083	3
H37Rv+PasA+RMP	1	0.15	3
H37Rv+INH	1.433333	0.057735	3
H37Rv+PasA+INH	0.916667	0.11547	3
H37Rv+RMP+INH	0.733333	0.057735	3
H37Rv+PasA+RMP+INH+	0.22	0.086603	3

Figure C5

	TNF		
	Mean	SD	N
H37Rv	1876.706	15.2828	3
H37Rv+PasA	3787.49	71.21859	3
H37Rv+RMP	3134.549	45.78547	3
H37Rv+PasA+RMP	4961.02	78.33327	3

Figure D5

	IL-10		
	Mean	SD	N
H37Rv	1629.704	66.66666	3
H37Rv+PasA	1014.889	151.2635	3
H37Rv+RMP	214.8889	33.945	3
H37Rv+PasA+RMP	37.11111	71.43445	3

Figure E5

	NO		
	Mean	SD	N
H37Rv	16.72489	0.3127151	3
H37Rv+PasA	41.09863	1.719933	3
H37Rv+RMP	18.98172	1.180474	3
H37Rv+PasA+RMP	16.09298	1.491556	3

Figure B6

	Noninfection LC3 dot		
	Mean	SD	N
Non-infection	0	0	3
RMP	3.703704	6.415003	3
Pas A	5.555555	9.622504	3
PasA+RMP	4.166667	7.216878	3

	Infection LC3 dot		
	Mean	SD	N
H37Rv	45.77778	4.811252	3
H37Rv+RMP	39.19312	4.127048	3
H37Rv+PasA	61.46561	5.040359	3
H37Rv+PasA+RMP	92.33334	8.333333	3

Figure A7

		CFU		
		Mean	SD	N
-RMP	H37Rv	2.57	0.257	3
	H37Rv+PasA	1.29	0.16125	3
	U0126+H37Rv	3.88	0.485	3
	U0126+H37Rv+PasA	3.99	0.57	3
	Bay+H37Rv	4.96	0.826667	3
	Bay+H37Rv+PasA	5.31	0.758571	3
	3-MA+H37Rv	4.1	0.455556	3
	3-MA+H37Rv+PasA	4.3	0.716667	3

+RMP	H37Rv			
	H37Rv+PasA	1.33	0.266	3
	U0126+H37Rv	0.73	0.121667	3
	U0126+H37Rv+PasA	2.9	0.725	3
	Bay+H37Rv	3.1	0.31	3
	Bay+H37Rv+PasA	3.3	0.55	3
	3-MA+H37Rv	2.8	0.35	3
	3-MA+H37Rv+PasA	4	0.444444	3
		3.8	0.633333	3

Figure B7

		TNF		
		**Mean**	**SD**	**N**
-RMP	H37Rv	1954.673	400.7709	3
	H37Rv+PasA	4224.02	503.1779	3
	U0126+H37Rv	2034.007	183.6083	3
	U0126+H37Rv+PasA	2228.673	279.4948	3
	Bay+H37Rv	70.67333	32.39341	3
	Bay+H37Rv+PasA	81.34	30.74627	3
	3-MA+H37Rv	2673.34	119.5715	3
	3-MA+H37Rv+PasA	3605.34	260.5405	3
+RMP	H37Rv	2932.01	200.3855	3
	H37Rv+PasA	5491.226	387.0599	3
	U0126+H37Rv	2644.209	367.2166	3
	U0126+H37Rv+PasA	3120.143	558.9896	3
	Bay+H37Rv	72.79353	35.63276	3
	Bay+H37Rv+PasA	89.474	20.49752	3
	3-MA+H37Rv	3020.874	239.1429	3
	3-MA+H37Rv+PasA	4290.355	338.7026	3

Figure C7

		IL-10		
		**Mean**	**SD**	**N**

-RMP	H37Rv	1837.556	139.2972	3
	H37Rv+PasA	1237	251.1216	3
	U0126+H37Rv	1506.444	270.0669	3
	U0126+H37Rv+PasA	1347.556	367.2773	3
	Bay+H37Rv	119.7778	50	3
	Bay+H37Rv+PasA	153.1111	101.3794	3
	3-MA+H37Rv	1169.778	204.8034	3
	3-MA+H37Rv+PasA	1325.333	277.0546	3
+RMP	H37Rv	459.3889	142.64	3
	H37Rv+PasA	199.473	83.9291	3
	U0126+H37Rv	753.2222	132.5333	3
	U0126+H37Rv+PasA	1036.581	234.1111	3
	Bay+H37Rv	155.7111	34.1339	3
	Bay+H37Rv+PasA	183.7333	55.41092	3
	3-MA+H37Rv	602.5778	245.2889	3
	3-MA+H37Rv+PasA	331.3333	169.7778	3

Figure D7

		NO		
		**Mean**	**SD**	**N**
-RMP	H37Rv	22.22611	0.699301	3
	H37Rv+PasA	47.3027	1.926803	3
	U0126+H37Rv	15.75991	2.624319	3
	U0126+H37Rv+PasA	21.41026	0.807483	3
	Bay+H37Rv	1.363636	0.201871	3
	Bay+H37Rv+PasA	1.596737	0.925088	3
	3-MA+H37Rv	23.39161	0.5341	3
	3-MA+H37Rv+PasA	32.25108	0.4995	3
+RMP	H37Rv	26.2	3.007	3
	H37Rv+PasA	21.354	2.441	3
	U0126+H37Rv	20.531	1.0645	3
	U0126+H37Rv+PasA	22.224	0.1503	3
	Bay+H37Rv	1.13053	0.201871	3
	Bay+H37Rv+PasA	1.01398	0.201871	3
	3-MA+H37Rv	22.2324	0.8214	3
	3-MA+H37Rv+PasA	21.1465	1.13024	3

Figure S3A

	% of Necrotic cells		
	**Mean**	**SD**	**N**

Non-infection	2.49	0.226274	2
PasA	1.09	0.19799	2
H37Rv	5.895	0.007071	2
H37Rv+PasA	2.49	0.226274	2

	**% of late apoptotic cells**		
	**Mean**	**SD**	**N**
Non-infection	9.06	0.282843	2
PasA	6.645	0.572756	2
H37Rv	26.8	1.230366	2
H37Rv+PasA	14.305	4.221427	2

Figure S3B

	**LDH release**		
	**Mean**	**SD**	**N**
Non-infection	1	0.01243	3
PasA	0.942118	0.02	3
H37Rv	57.45813	2.212917	3
H37Rv+PasA	37.67488	3.884199	3

Figure S3C

	**CFU**		
	**Mean**	**SD**	**N**
H37Rv	1.466667	0.23094	3
H37Rv+PasA	0.8	0.2	3
H37Rv+RMP	0.733333	0.23094	3
H37Rv+PasA+RMP	0.266667	0.11547	3
